# Oligometastases of Esophageal Squamous Cell Carcinoma: A Review

**DOI:** 10.3390/cancers16040704

**Published:** 2024-02-07

**Authors:** Yuta Sato, Yoshihiro Tanaka, Ryoma Yokoi, Hiroshi Tsuchiya, Yuki Sengoku, Masahiro Fukada, Itaru Yasufuku, Ryuichi Asai, Jesse Yu Tajima, Shigeru Kiyama, Takazumi Kato, Katsutoshi Murase, Nobuhisa Matsuhashi

**Affiliations:** 1 Department of Gastroenterological Surgery and Pediatric Surgery, Gifu University Graduate School of Medicine, 1-1 Yanagido, Gifu City 501-1194, Gifu Prefecture, Japan; 2 Department of Clinical Anatomy Development Studies, Gifu University Graduate School of Medicine, 1-1 Yanagido, Gifu City 501-1194, Gifu Prefecture, Japan

**Keywords:** esophageal cancer, squamous cell carcinoma, oligometastases, oligo-recurrence, sync-oligometastases, systemic therapy, local therapy, multimodal therapy, distant metastases, conversion surgery

## Abstract

**Simple Summary:**

A review was conducted to examine treatment strategies for oligo-recurrence with a controlled primary lesion and sync-oligometastases with an active primary lesion in esophageal squamous cell carcinoma. Although oligo-recurrence cases with possible therapeutic interventions had better outcomes, there may be selection bias due to retrospective studies. However, it is very clear that a small number of cases of oligometastases can be cured by local therapy, including surgical resection of metastases and radical chemoradiotherapy. The results of several ongoing clinical trials may lead to new treatment strategies for patients with oligometastases from esophageal squamous cell carcinoma in the future.

**Abstract:**

Patients with oligometastases show distant relapse in only a limited number of regions. Local therapy such as surgical resection, radiotherapy, chemoradiotherapy, and radiofrequency ablation for the relapsed sites may thus improve patient survival. Oligometastases are divided into oligo-recurrence and sync-oligometastases. Oligo-recurrence indicates a primary lesion that is controlled, and sync-oligometastases indicate a primary lesion that is not controlled. The management of oligo-recurrence and sync-oligometastases in esophageal squamous cell carcinoma has not been clearly established, and treatment outcomes remain equivocal. We reviewed 14 articles, including three phase II trials, that were limited to squamous cell carcinoma. Multimodal treatment combining surgical resection and chemoradiotherapy for oligo-recurrence of esophageal squamous cell carcinoma appears to be a promising treatment. With the development of more effective chemotherapy and regimens that combine immune checkpoint inhibitors, it will become more likely that sync-oligometastases that were unresectable at the initial diagnosis can be brought to conversion surgery. Currently, a randomized, controlled phase III trial is being conducted in Japan to compare a strategy for performing definitive chemoradiotherapy and, if necessary, salvage surgery with a strategy for conversion surgery in patients who can be resected by induction chemotherapy.

## 1. Introduction

Worldwide, esophageal cancer is the fifth most common cause of cancer-related death for men and the eighth for women [1], and although relatively rare, it remains a significant health problem worldwide. Esophageal cancer is more common in men than women and tends to occur in elderly people [2]. The geographical difference in the incidence rate and prognosis of esophageal cancer is significant between the two most common histological subtypes: squamous cell carcinoma and adenocarcinoma [2]. Squamous cell carcinoma of the esophagus is also common in limited areas, for example, Eastern Asia and parts of Africa. Squamous cell carcinoma is more common in developing countries and is often associated with alcohol and tobacco use. Adenocarcinoma is more common in developed countries and is often associated with obesity and gastroesophageal reflux disease. The prognosis of esophageal cancer depends on various factors, including the stage of the cancer and the individual’s overall health. However, early detection and treatment can improve outcomes. The American Cancer Society provides the following estimated 5 year relative survival rates for esophageal cancer based on stage: approximately 49% of patients with localized esophageal cancer (cancer is only in the esophagus), 26% of people with regional esophageal cancer (cancer has spread to nearby lymph nodes or tissues), and 5% of people with distant esophageal cancer (cancer has spread to distant parts of the body) survive for at least 5 years after diagnosis [3].

Preoperative chemotherapy for locally advanced esophageal cancer is the current standard of care in Japan [4,5]. The JCOG1109 trial showed that triplet docetaxel, cisplatin, and 5-fluorouracil (DCF) chemotherapy resulted in significantly better overall survival (OS) and progression-free survival (PFS) compared with cisplatin and 5-fluorouracil, and DCF is the standard neoadjuvant treatment [6]. In Europe and the United States, the standard treatment for locally advanced esophageal carcinoma is surgery after neoadjuvant chemoradiotherapy with paclitaxel plus carboplatin, based on the CROSS trial [7], and a prospective randomized controlled multicenter phase III trial comparing perioperative chemotherapy (FLOT protocol) to neoadjuvant chemoradiation (CROSS protocol) in patients with esophageal adenocarcinoma is ongoing (ESOPEC: NCT 02509286, Neo-AEGIS: NCT 01726452) [8,9]. Surgery performed after controlling local and systemic micrometastases with preoperative induction chemotherapy has improved radicality and prolonged prognosis [10]. Metastatic esophageal cancer, however, is generally considered a non-surgical disease because of the non-curative nature of this morbid condition. The treatment strategy for these patients generally involves treatment with palliative intent that prioritizes symptom control over more aggressive treatment.

**Figure 1:**
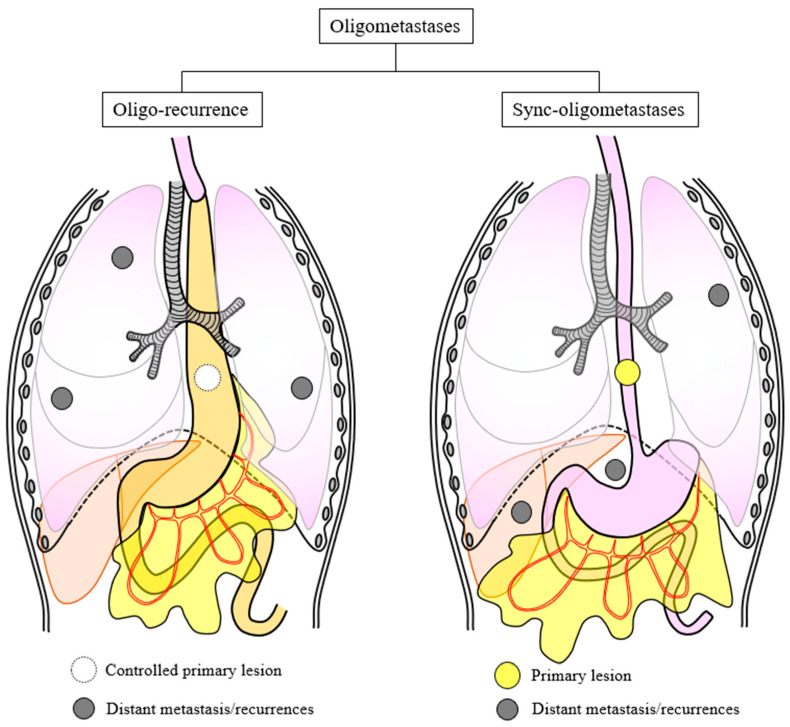
Illustration representing the concept of oligometastases in esophageal squamous cell carcinoma: oligo-recurrence, with a controlled primary lesion (**left**), and sync-oligometastases, with an active primary lesion (**right**).

A population-based study reporting on 3876 patients with esophageal or gastric adenocarcinoma or squamous cell carcinoma between 1990 and 2017 in The Netherlands revealed that the most common metastatic locations for this cancer were the liver (56%), extra-regional lymph nodes (53%), and lung (50%) [11]. Esophageal adenocarcinoma more frequently metastasizes to the peritoneum and bone in comparison with esophageal squamous cell carcinoma [11]. In contrast, lymph node metastasis is the most common form of recurrence of esophageal squamous cell carcinoma (42%), followed by lung (15%) and liver (13%) [12]. In the JCOG9907 trial, of 157 cases of recurrence, 44 cases (28%) were of regional metastases (local and regional lymph nodes), 25 cases (16%) were of distant metastases and regional lymph nodes, and 86 cases (55%) were of distant metastases only [10]. Lungs were the most common metastatic organ (15%), followed by liver (13%) and bone (10%) [10]. Most of the recurrences appeared within 3 years after surgery. In the JCOG1109 trial that followed the JCOG9907 trial, of 252 recurrent cases, 88 cases (35%) were of regional recurrence (local and regional lymph nodes), 66 cases (26%) were of distant metastases and regional lymph node metastases, and 98 cases (39%) were of distant metastases only, and as in the JCOG9907 trial, most recurrent cases appeared within 3 years after surgery [6]. The cancer recurrence or metastasis has been considered to occur in the final stage of the patient’s life. From this perspective, even if the site of recurrence or metastases is a single site, the cancer can be disseminated hematogenously or lymphatically throughout the body, which means that local therapy cannot eradicate all cancer cells. In recent years, importance has been placed on distinguishing limited metastatic disease, or oligometastatic disease, from extensive metastatic disease. The general concept of oligometastatic cancer was first introduced in 1995 and described a clinical state between locally confined and systemic metastasized disease [13]. According to the European Society for Radiotherapy and Oncology (ESTRO) and American Society for Radiation Oncology (ASTRO) consensus document, oligometastatic disease has been defined as one to five metastatic lesions that can be safely treated with or without a controlled primary tumor [14]. Oligometastatic cancer, an intermediate stage before widespread metastasis, does not immediately spread widely and can benefit from a multidisciplinary treatment [15]. However, given that a certain percentage of cancer patients progress rapidly after initial treatment, rational stratification improves the recognition of the biological behavior of oligometastatic cancer. In recent years, oligometastases have been classified into two statuses (Figure 1). The first is oligo-recurrence, which refers to cancer patients with 1 to 5 recurrences that are treated by local therapy, such as surgical resection, radiotherapy, chemoradiotherapy, and radiofrequency ablation, and who have a controlled primary lesion. The second is called sync-oligometastases and refers to cancer patients with 1 to 5 recurrences and an active primary lesion [16,17,18,19]. Sync-oligometastases are a concept first proposed by Niibe et al. [18]. They reported that oligo-recurrence can predict a satisfactory prognosis of only brain oligometastases in patients with non-small cell lung cancer (NSCLC) treated with stereotactic radiosurgery or stereotactic radiotherapy, and they investigated the importance of oligo-recurrence compared with sync-oligometastases in patients with brain-only NSCLC oligometastases [18]. Furthermore, Niibe et al. used this oligometastases concept to examine 1378 patients with pulmonary oligometastases treated by stereotactic body radiation therapy (SBRT) [19]. They confirmed the superior OS after pulmonary oligo-recurrence compared to pulmonary sync-oligometastases in a large nationwide study. As a result, 3 year OS was 64.0% for oligo-recurrence and 47.5% for sync-oligometastases (*p* < 0.001) [19]. They also proposed that simultaneous recurrence of primary and metastatic lesions is closer to sync-oligometastases than oligo-recurrence. The concept of oligo-recurrence was proposed before that of metachronous oligometastases. Thus, oligo-recurrence was the original concept related to oligometastases with a controlled primary lesion [18].

The guidelines for the management of oligo-recurrence and sync-oligometastases of esophageal squamous cell carcinoma are, however, not clearly established, and survival outcomes remain unclear. Thus, the aim of this review was to assess current practice for the treatment of oligometastatic esophageal squamous cell carcinoma.

## 2. Materials and Methods

A search was performed and last updated 16 September 2023, in PubMed with the keywords “squamous cell carcinoma” and “oligometastases” or “oligo-recurrence” or “sync-oligometastases”. The selection was limited to articles on esophageal squamous cell carcinoma, excluding head and neck cancer and lung cancer, and to articles published only in English.

Because of differences in prognosis, recurrence format, chemotherapy regimens, and effectiveness of radiotherapy between squamous cell carcinoma and adenocarcinoma [10,11,12], this review focused on studies in which the majority of cases were of squamous cell carcinoma and excluded studies with a high adenocarcinoma content (squamous cell carcinoma content was defined as 93% or higher). We also excluded gray literature (such as conference abstracts and clinical trial registries).

## 3. Results

The study of oligometastatic cancers is still relatively new, with limited data available specifically regarding esophageal squamous cell carcinoma. Our search ultimately retrieved 14 articles, including three phase II trials (Table 1). As there was variation in the definition of the term oligometastases in each of the articles, the classification was made using the terms oligo-recurrence and sync-oligometastases proposed by Niibe et al. [16,17,18,19]. As the number of metastases and the number of metastatic organs defining the oligometastases also varied, they were extracted and listed (Table 1).

### 3.1. Surgical Resection for Oligometastatic Esophageal Squamous Cell Carcinoma

There were two studies comparing surgical resection and non-resection of oligo-recurrences. Tsai et al. retrospectively studied patients with oligo-recurrence of esophageal cancer after radical esophagectomy and analyzed prognostic factors for OS and post-recurrence survival (PRS), as well as the impact of surgical resection on survival [32]. As a result, the significant prognostic factors of PRS with poor outcome included mediastinal lymph node recurrence and pathologic T3 stage, and compared with non-surgery, surgery for resectable recurrence could achieve better PRS for patients with no comorbidities (hazard ratio [HR]: 0.36, 95% confidence interval [CI]: 0.14–0.94, *p* = 0.038).

### 3.2. Radiation or Chemoradiation for Oligometastatic Esophageal Squamous Cell Carcinoma

In a multi-institutional retrospective study, Yamashita et al. compared treatment outcomes following chemoradiation and radiation alone for oligo-recurrence [20]. The results showed a 3 year OS rate of 39.7% (95% CI: 32.1–47.3%) with chemoradiation and 20.8% (95% CI: 8.3–37.0%) with radiation alone (*p* = 0.000055). In a retrospective study of 239 patients with oligo-recurrence, Li et al. reported that patients receiving chemoradiation were associated with improved rates of PFS (9.5 vs. 3.8 months, *p* < 0.001) and OS (21.3 vs. 12.7 months, *p* < 0.001) compared to chemotherapy alone [27]. Lin et al. reported that radical, definitive re-irradiation may lead to longer survival in patients with oligo-recurrence after previous curative radiotherapy for esophageal squamous cell carcinoma (their 5 year OS rate was 21%) [23].

### 3.3. Treatment Strategy for Sync-Oligometastases of Esophageal Squamous Cell Carcinoma

Chen et al. reported in 2019 a retrospective study of 461 stage IVB patients with sync-oligometastases of squamous cell carcinoma [22]. In their study, sync-oligometastases were defined as three or fewer distant metastatic lesions. The definitive chemoradiotherapy group (*n* = 196) (50 Gy to the primary tumor and 45 Gy to all sync-oligometastases) had a statistically significantly better PFS than the chemotherapy alone group (*n* = 265) (8.7 months vs. 7.3 months, respectively, *p* = 0.002). On the other hand, OS tended to be higher in the definitive chemoradiation group (16.8 months vs. 14.8 months, respectively, *p* = 0.056). A recent large retrospective study reported by Shi et al. in 2022 examined definitive dose-concurrent chemoradiotherapy (50 Gy to the primary tumor and 45 Gy to the metastatic site) in patients with oligometastatic squamous cell carcinoma histology [31]. In their study, sync-oligometastases were defined as five or fewer distant metastatic lesions. They reported significantly improved OS (median 18.5 months) and PFS (median 9.7 months) in the chemoradiotherapy group (*n* = 240) compared to the chemotherapy alone group (*n* = 292) (*p* < 0.001, *p* < 0.001, respectively).

### 3.4. Phase II Non-Randomized Trials for Oligometastatic Esophageal Squamous Cell Carcinoma

Three phase II non-randomized trials were included in this systematic review of esophageal squamous cell carcinoma only. Liu et al. conducted a prospective, single-arm, phase II trial on the safety and efficacy of SBRT for patients with oligo-recurrence (three or fewer metastases) [24]. In this study, 34 patients with 40 oligo-recurrence lesions, including 25 in distant organs and 15 in nonregional lymph nodes, were treated with SBRT. The median PFS time was 13.3 months (95% CI: 10.7–15.9 months), and the 1 and 2 year PFS rates were 55.9% and 33.8%, respectively. Furthermore, Liu et al. are currently conducting the ESO-Shanghai 13 trial, a prospective, multicenter, randomized, phase II trial to assess the impact of combined local treatment (such as radiotherapy, surgery, and thermal ablation) and chemotherapy for patients with esophageal squamous cell carcinoma [30]. The definition of oligo-recurrence in this trial is four or fewer metastases. All patients will be randomized and receive either chemotherapy alone or chemotherapy plus local treatment with the same probability.

## 4. Discussion

For a long time, there has been no common definition or standard treatment approach for oligometastases from esophageal cancer. However, the concept of oligometastases is becoming increasingly recognized in esophageal squamous cell carcinoma. Oligometastases, as proposed by Niibe et al., are classified into oligo-recurrence, in which the primary tumor is controlled, and sync-oligometastases, in which the primary tumor is uncontrolled [16,17,18,19]. There have also been several studies of other organs using this definition. Yamashita et al. showed that the median OS of patients with lung oligo-recurrence was significantly higher than that of patients with sync-oligometastases (66.6 vs. 23.9 months, *p* = 0.0029), and by multivariate analysis, sync-oligometastases and multiple oligometastatic tumors were significant unfavorable factors for both OS and relapse-free survival [34].

In cases of recurrence after radical esophageal cancer resection, some areas may be difficult to resect or may cause serious adverse events, and the patient’s general condition at the time of recurrence also affects treatment options. Therefore, there are few reports of treatment outcomes in large numbers of patients with various pathologies, and the evidence showing that aggressive treatment leads to improved prognosis and quality of life in patients with recurrent disease is currently insufficient. However, several observational studies have reported the efficacy of curative-seeking surgical resection for localized metastatic recurrence after radical esophageal cancer resection, that is, oligo-recurrence.

In a study of 206 cases of recurrence after initial curative treatment in which 119 cases were classified as oligo-recurrence, Ohkura et al. reported a 5 year OS rate of 55.6% with resection [28]. Kudou et al. reported that the 5 year OS rate after resection of recurrent lesions for oligo-recurrence was significantly better than that for lymph node metastasis (39.5%), lung metastasis (54.5%), and another organ resection (27.9%) [35]. Multivariate analysis showed that patients with pathological N0-1 at initial surgery, pulmonary metastases, a postoperative time to recurrence of 550 days or more, R0 resection, and no serious complications after resection of the recurrence had a good prognosis. Watanabe et al. reported that the 3 year OS rate for surgical resection of lymph node recurrence after radical resection was approximately 76%, with cervical lymph node recurrence in particular having a greater effect on resection than mediastinal or abdominal lymph node recurrence [36]. Especially for cervical lymph nodes, Ma et al. also reported that resection had resulted in a significantly better prognosis than that with radiotherapy or chemoradiotherapy [37]. Wang et al. reported that in recurrence confined to the cervical lymph nodes, multivariate analysis showed a good prognosis for the number of lymph node metastases (two or fewer) at the time of radical esophageal cancer resection [38]. In most of these reports, however, the criteria for selecting treatment methods are not clear, and individual decisions are made based on the number and distribution of recurrence sites, as well as the general condition of the patients. It should also be recognized that the data were obtained by selecting treatment methods considered possible for cases for which active treatment was determined to be possible in the first place. Thus, there is likely to be significant bias in background factors in the comparison of outcomes between treatments.

Sync-oligometastases, in contrast, refer to cStage IVB in the Japanese classification of esophageal cancer, which indicates that the cancer has progressed beyond the local area and that systemic treatments such as chemotherapy and chemoradiation are recommended. In these cases, the development of therapies combining immune checkpoint inhibitors (ICIs) and anticancer agents has been underway in recent years. The results of the ATTRACTION-3 study [39], ESCORT study [40], and KEYNOTE-181 study [41] showed that the anti-programmed cell death receptor 1 antibody was associated with significant improvement in OS in previously treated patients with advanced esophageal cancer compared with chemotherapy and might become a new standard secondary treatment option. The rates of Grade 3 or higher treatment-related adverse events in each trial were 18% in the nivolumab group [39] and 18% in the pembrolizumab group [41].

In the KEYNOTE-590 study of advanced or recurrent esophageal cancer, pembrolizumab was added to the conventional standard chemotherapy regimen of cisplatin plus fluorouracil, and the median OS was 13.9 months in the combination group compared with 8.8 months in the placebo group, indicating a significant difference (HR: 0.57, 95% CI: 0.43–0.75, *p* < 0.0001) [42,43,44]. Adverse events were slightly increased in the pembrolizumab-chemotherapy combination group but were considered acceptable [42]. In the CheckMate 648 study, similar patients were compared among those receiving standard therapy versus nivolumab plus standard therapy versus nivolumab plus ipilimumab. The results showed that the median OS was 15.4 months (95% CI: 11.9–19.5 months) in the nivolumab plus standard therapy group, 13.7 months (95% CI: 11.2–17.0 months) in the nivolumab plus ipilimumab group, and 9.1 months (95% CI: 7.7–10.0 months) in the standard therapy group, all significantly higher [45,46,47]. Adverse events in the nivolumab plus standard chemotherapy group tended to be more frequent than those in the nivolumab plus ipilimumab and chemotherapy alone groups but were considered acceptable [45]. Grade 3 or higher liver toxicity occurred in 2% of patients in the nivolumab plus chemotherapy group. These regimens are now considered the standard therapy for squamous cell esophageal cancer in Japan. Additionally, a new multicohort phase I study (the JCOG1804E trial) is currently underway to evaluate the safety and efficacy of neoadjuvant nivolumab in combination with chemotherapy for esophageal squamous cell carcinoma in Japan [48]. The primary endpoint of this trial, called the FRONTiER trial, is the incidence of dose-limiting toxicities from the initial dose to postoperative day 30. The secondary endpoint included adverse events during the perioperative period and at 30 days, as well as the objective response rate, histopathological complete response rate, and R0 resection rate.

Zhao et al. conducted a phase II non-randomized trial that evaluated the efficacy and safety of low-dose radiation therapy plus immunotherapy and second-line chemotherapy for patients with oligometastatic esophageal squamous cell carcinoma [33]. In this trial, patients with oligometastatic esophageal squamous cell carcinoma who had failed first-line ICIs plus chemotherapy were treated with low-dose radiation therapy plus camrelizumab and second-line irinotecan chemotherapy. Currently, no trials in oligometastatic recurrence have compared ICIs with conventional chemotherapy or local therapy such as surgery or radiation. Furthermore, there is no evidence for the combination of chemoradiotherapy and ICIs. Therefore, the results of their trial will greatly contribute to future treatment development. For advanced esophageal squamous cell carcinoma, especially locally advanced disease, surgery followed by neoadjuvant chemotherapy plus ICIs or chemoradiotherapy combined with ICIs should be further explored, but current clinical studies are limited to phase I-II trials and lack long-term follow-up data. In lesions that respond to high-dose local radiation, a systemic antitumor effect may be observed, and nonirradiated distant tumor sites may regress [49]. This phenomenon, called the abscopal effect, was first reported by Mole in 1953 [50]. With the development of immunotherapy in recent years, the number of publications has increased rapidly. This effect has been reported more frequently in malignancies such as lymphoma, melanoma, and renal cell carcinoma [51]. However, data on metastatic esophageal cancers are scarce. This ICI-related effect will be further investigated in the future and may become a new treatment strategy.

Along with the development of highly effective chemotherapies or chemoradiotherapy, an increasing number of sync-oligometastases cases that were considered unresectable at the time of initial diagnosis are becoming resectable as a result of initial treatment. In such cases, when further chemotherapy or chemoradiotherapy alone is expected to limit reduction, surgical treatment is called conversion surgery in the hope that R0 resection (complete response [CR]) may be achieved. Conversion therapy is defined as surgery aiming to cure after initial treatment for tumors that were initially unresectable due to adjacent organ invasion or distant metastases [52]. In general, patients undergoing conversion surgery can be expected to have a favorable long-term prognosis when R0 resection is obtained, but at the same time, the frequency of postoperative complications increases. As systemic treatment developed, more attention was directed toward conversion surgery. In a Japanese multicenter phase II study (COSMOS study), based on the efficacy evaluation after induction chemotherapy for unresectable advanced esophageal cancer, resectable patients were treated with surgery, and unresectable patients were treated with chemoradiotherapy, with conversion surgery performed if resectable at a chemoradiation dose of 40 or 60 Gy. Surgical treatment was performed in 42% of cases (20/48 cases), and the R0 resection rate was 95% (19/20). Only one patient underwent R1 resection after irradiation with 60 Gy. The histopathological efficacy of Grade 2 or higher was 60% (12/20 cases). Among the 17 patients who progressed to chemoradiotherapy, the CR rate was 23.5% (4/17). In the end, 19 patients underwent R0 resection after DCF therapy, and 4 patients who transitioned to chemoradiotherapy obtained CR. In total, 47.9% (23/48) of the patients were judged to have substantial CR. The OS rates at 1 and 3 years for the 19 patients who underwent R0 resection were 100% and 71.4%, respectively, and the 1 and 3 year PFS rates were 83.6% and 61.3%, respectively [53,54,55,56]. Compared to surgery for resectable esophageal cancer, although in-hospital mortality and complications are slightly higher, they are considered acceptable if the patient can tolerate the surgery, given the lack of other effective treatment options and the relatively good outcome of R0 resection. Based on these results, a randomized, controlled, phase III trial (JCOG1510) is currently being conducted in Japan to compare the strategy for performing definitive chemoradiotherapy and, if necessary, salvage surgery with the strategy for conversion surgery in patients who can be resected by induction chemotherapy [57,58]. In total, 230 patients will be accrued from 47 Japanese institutions over 4.5 years. The primary endpoint is OS, and the secondary endpoints are PFS, CR rate of chemoradiotherapy, response rate of DCF, adverse events of DCF and chemoradiotherapy, late adverse events, and surgical complications. Overseas, Bedenne et al. compared outcomes in two groups of patients with resectable, locally advanced squamous cell carcinoma of the thoracic esophagus who responded to chemoradiotherapy: those who underwent conversion surgery and those who continued chemoradiotherapy (the FFCD 9102 trial) [59]. Median survival time was better in the chemoradiotherapy group, at 17.7 and 19.3 months, respectively (HR = 0.90, *p* = 0.49). In addition, at 3 months after treatment, the mortality rate due to perioperative complications in the conversion surgery group was 9.3%, which was significantly higher than that of 0.8% in the group that continued chemoradiotherapy (*p* = 0.002). As a result, it would appear desirable not to perform conversion surgery on patients who have had a significant response to chemoradiotherapy. Conversely, the safety of conversion surgery after chemoradiotherapy was reported by Stahl et al. [60]. They conducted a phase III trial comparing chemoradiotherapy followed by conversion surgery versus chemoradiotherapy alone for locally advanced squamous cell carcinoma of the thoracic esophagus. The median survival time was slightly better in the conversion surgery group (16.4 vs. 14.9 months, respectively), and local control was significantly better in the conversion surgery group (HR = 2.1, *p* = 0.003). However, treatment-related deaths were significantly higher in the conversion surgery group (12.8% vs. 3.5%, *p* = 0.03). The results of these two trials suggest that surgery after chemoradiation for locally advanced esophageal cancer may be unsafe because it leads to increased treatment-related deaths.

The analysis of prognostic factors is also important. The identification of poor prognostic factors for the local treatment of oligo-recurrence will provide useful information for the development of postoperative chemotherapy. Molecular residual disease evaluated by circulating tumor DNA (ctDNA) may be another promising prognostic measure for recurrence after the surgical resection of oligo-recurrence or sync-metastasis. There are unmet needs for prognostic biomarkers for dynamically monitoring disease progression and detecting minimal residual disease. Although there is very little evidence at this time for correlations between survival outcomes and ctDNA in esophageal cancer [61], being positive for ctDNA in resectable colorectal cancer confirmed by liquid biopsy is a robustly poor prognostic factor for postoperative recurrence of disease [62]. The usefulness of ctDNA in esophageal squamous cell carcinoma, including oligo-recurrence or sync-oligometastases, remains to be investigated in the future. Further discussions and analyses are needed to establish a standard treatment for oligometastases from esophageal cancer.

The review has several potential limitations. First, it was limited to the searching of one database (PubMed), which may cause publication bias. Second, papers not written in English were excluded. Third, publication bias exists because gray literature (such as conference abstracts and clinical trial registries) was excluded.

## 5. Conclusions

The majority of reports on cases of oligo-recurrence after radical resection of esophageal cancer are about cases judged to be treatable because of the wide variety of factors that influence the choice of treatment, including the form, extent, and number of recurrences and the general condition of the patient at the time of recurrence. Therefore, it is difficult at this point to formulate a definitive opinion regarding whether to aggressively treat oligo-recurrence cases with the aim of curing the disease. Nevertheless, it is very clear that there are a small number of cases in which a radical cure can be achieved by resection of metastases and chemoradiotherapy, and many reports of observational studies support this. A large-scale prospective study at multiple centers is desired in the future. For sync-oligometastasis cases that are unresectable, conversion surgery may provide long-term survival in cases of local disease persistence or recurrence after chemoradiotherapy. However, it must be borne in mind that the frequency of postoperative complications and the rate of in-hospital mortality are higher. Based on the results of this review, at the current stage, oligo-recurrence appears to have a better prognosis than synch-oligometastases. Ongoing prospective studies will reveal whether perioperative chemotherapy combined with surgery can prolong the survival outcomes of patients with various localized metastases in the future. Future advances in genomic medicine will make use of new biomarkers such as circulating tumor deoxyribonucleic acid, which will have a significant impact on treatment decisions in cases of oligometastases of esophageal squamous cell carcinoma. The development of new treatment strategies, including new chemotherapeutic agents or regimens, could improve the survival outcomes of patients with oligometastasis from esophageal squamous cell carcinoma. For further developments to occur, it is important to understand the comprehensive characteristics and prognosis and to provide a framework for the integrated definition, classification, and treatment of oligometastatic esophageal cancer.

## Figures and Tables

**Table 1:** Results of a review limited to oligometastases of esophageal squamous cell carcinoma.

Authors	Year	Country	Inclusion			Patients	Oligometastasis		Histology		Treatment	Key Results
			Type	Center	Period		Type	Definition	SCC	Other		
Yamashita [20]	2017	Japan	RNR	Multi	2000–2015	237	OR	<5 lymph node	231	6	CRT vs. RT	3 year OS: 36.7% vs. 20.8% (*p* = 0.00005)
Hamai [21]	2018	Japan	RNR	Single	1990–2013	133	OR	ns	133	0	Various	Median DFI: 9.1 m
Chen [22]	2019	China	RNR	Multi	2012–2015	461	SO	<3 metastases	446	15	CRT vs. RT	DCR: 81.6 vs. 64.5% (*p* < 0.001)
Lin [23]	2020	Taiwan	RNR	Multi	2012–2018	30	OR	<5 metastases	30	0	Re-irradiation	5 year OS: 21%
Liu [24]	2020	China	P2 NR	Single	2015–2018	34	OR	<3 metastases	34	0	RT	Median PFS: 13.3 m
Morinaga [25]	2020	Japan	RNR	Single	2005–2019	43	OR	<5 metastases (single organ)	43	0	CLT vs. CT	5 year OS: 39.2 vs. 14.4 m (*p* = 0.038)
Yamashita [26]	2020	Japan	RNR	Single	2012–2017	18	OR	<5 cm, <3 lesions	18	0	RT	OS: SCC < other cancers
Li [27]	2020	China	RNR	Single	ns	239	OR	<5 metastases <3 lesions	239	0	CRT vs. CT	OS: 21.3 vs. 12.7 m (*p* < 0.001) PFS: 9.5 vs. 3.8 m (*p* < 0.001)
Ohkura [28]	2020	Japan	RNR	Single	2011–2017	119	OR	<5 metastases (single domain)	ns	ns	SR vs. non	3 year OS: 64.3% vs. 9.8% 5 year OS: 55.6% vs. 0%
Li [29]	2021	China	RNR	Single	2009–2018	82	OR	<5 metastases	78	4	RT vs. non	Median OS: 14 vs. 7 m (*p* = 0.0016)
Liu [30]	2021	China	P2 NR	Single	2019–2022	102	OR	<4 metastases <3 (single organ)	102	0	RT	On going
Shi [31]	2022	China	RNR	Multi	2012–2018	532	SO	<5 metastases	532	0	CRT vs. CT	OS: 18.5 m vs. 15.2 m (*p* < 0.001) PFS: 9.7 m vs. 7.6 m (*p* < 0.001)
Tsai [32]	2022	Taiwan	RNR	Single	2004–2017	63	OR	<5 metastases	60	3	SR vs. non	3 year PRS rate: 42.9% vs. 23.5%
Zhao [33]	2023	China	P2 NR	Multi	2018–2021	49	SO	<5 metastases	49	0	RT + ICI	PFS 6.9 m (95% CI 4.6–9.3) OS 12.8 m (95% CI 10.1–15.5)

*RNR* retrospective non-randomized trial, *P2 NR* phase 2 non-randomized trial, *ns* not specified, *OR* oligo-recurrence, *SO* sync-oligometastases, *SCC* squamous cell carcinoma, *CRT* chemoradiation therapy, *RT* radiation therapy, *CLT* combined local therapy (as chemotherapy with local therapy such as resection, radiation, and radiofrequency ablation), *CT* chemotherapy, *SR* surgical resection, *ICI* immune checkpoint inhibitor, *OS* overall survival, *DFI* disease-free interval, *m* month, *DCR* disease-control rate, *PFS* progression-free survival, *PRS* post-recurrence survival, *CI* confidence interval.
